# Changes of circulating tumor cells expressing CD90 and EpCAM in early-phase of atezolizumab and bevacizumab for hepatocellular carcinoma

**DOI:** 10.1016/j.heliyon.2024.e34441

**Published:** 2024-07-10

**Authors:** Takuto Nosaka, Yosuke Murata, Yu Akazawa, Kazuto Takahashi, Tatsushi Naito, Hidetaka Matsuda, Masahiro Ohtani, Yasunari Nakamoto

**Affiliations:** Second Department of Internal Medicine, Faculty of Medical Sciences, University of Fukui, Fukui, Japan

**Keywords:** Hepatocellular carcinoma, Circulating tumor cell, Atezolizumab, CD90, EpCAM

## Abstract

Circulating tumor cells (CTCs) are noninvasive biomarkers that can indicate the therapeutic response and prognosis. The study aimed to investigate the cellular characteristics of CTCs focusing on monitoring during atezolizumab and bevacizumab (Atezo-Bev) therapy in patients with hepatocellular carcinoma (HCC). Peripheral blood samples were collected from 10 healthy controls and 40 patients with HCC. CTCs enriched using RosetteSep™ Human CD45 depletion cocktail were analyzed by multiparametric flow cytometry. CTC isolation was based on PanCK(+)CD45(−) cells, and CTCs exhibiting markers CD90, CD133, EpCAM, or vimentin. The total number of CTCs and the number of CTCs expressing CD90, CD133, EpCAM, and vimentin were correlated with the BCLC stage of HCC. The change in total CTC count accurately reflected the initial response to Atezo-Bev therapy. The numbers and mean fluorescence intensity of the CTC subsets expressing CD90 and EpCAM molecules decreased in patients with partial response/stable disease, and increased in patients with progressive disease and were markedly correlated with overall survival. CD90(+) and EpCAM(+) CTCs may be candidate biomarkers for the early prediction of the treatment response and the overall survival of patients with HCC receiving Atezo-Bev therapy.

## Highlights

• CTCs were analyzed with multiparametric flow cytometry during Atezo-Bev therapy.
• CD90, CD133, EpCAM, and vimentin(+) CTCs correlated with the BCLC stage of HCC.
• Changes of CD90^−^and EpCAM(+) CTCs can predict early response of Atezo-Bev therapy.
• CD90^−^and EpCAM(+) CTCs represent biomarker of OS in HCC patients of Atezo-Bev.

## Abbreviations

AEs: adverse events
AFP: a-fetoprotein
ALBI: albumin-bilirubin
Atezo-Bev: atezolizumab and bevacizumab
BCLC stage: Barcelona Clinic Liver Cancer stage
CSC: cancer stem cell
CTC: circulating tumor cell
DCP: des-gamma-carboxy prothrombin
ECOG: Eastern Cooperative Oncology Group
EMT: epithelial-mesenchymal transition
ICI: immune checkpoint inhibitor
HAIC: hepatic arterial infusion chemotherapy
HBsAg: surface antigen of hepatitis B virus
HBV: hepatitis B virus
HCC: hepatocellular carcinoma
HCV Ab: anti-hepatitis C virus antibody
IQR: interquartile range
MFI: mean fluorescence intensity
NANOG: Nanog homeobox
NBNC: non-B-non-C
OS: Overall survival
PBS: phosphate buffered saline
PD: progressive disease
PFS: Progression-free survival
PR: partial response
PS: performance status
RECIST: Response Evaluation Criteria for Solid Tumors
SD: stable disease
SOX2: SRY-box transcription factor 2
TACE: transcatheter arterial chemoembolization

## 1 Introduction

Hepatocellular carcinoma (HCC) is a highly aggressive malignancy imposing a significant burden on health care systems worldwide [1]. Despite advances in treatment options, the prognosis of advanced HCC remains poor, emphasizing the need for new therapeutic strategies [2]. The combination of atezolizumab, an immune checkpoint inhibitor (ICI), and bevacizumab, a vascular endothelial growth factor inhibitor, has shown promising results in clinical trials as a first-line treatment for advanced HCC [3]. However, the identification of reliable biomarkers to predict treatment response and monitor disease progression in patients undergoing combination therapy remains an area of active research.

Circulating tumor cells (CTCs), which are shed from primary tumors and circulate in peripheral blood, represent a dynamic and heterogeneous population of tumor cells that can provide valuable information about tumor biology [4]. CTCs have emerged as potential non-invasive biomarkers for various solid tumors, providing insights into the treatment response and prognosis [5]. Additionally, CTCs offer a unique opportunity for real-time monitoring of treatment response, providing an early indication of therapy effectiveness or the need for treatment modifications [6]. In the context of HCC, the detection and characterization of CTCs have immense value, particularly during the course of treatment, as they can serve as real-time indicators of treatment response and disease dynamics [7]. By longitudinal monitoring CTC levels and molecular characteristics during the course of atezolizumab and bevacizumab combination therapy, clinicians can gain valuable insights into treatment efficacy and disease progression [8].

In this study, we investigated the molecular and cellular characteristics of CTC, with a special emphasis on its detection and monitoring during the course of atezolizumab and bevacizumab combination therapy in patients with HCC. The therapeutic effects were correlated with changes in the expression of CD90 and EpCAM molecules observed in CTCs at the early timepoints of treatment and reflected the long-term prognosis of HCC patients. These results demonstrate that non-invasive monitoring of molecular alterations in CTCs may contribute to the early detection of predicted therapeutic responses in patients with HCC treated with ICI combination therapy.

## 2 Patients and methods

### 2.1 Study protocol and patients

This retrospective study was performed in accordance with the Declaration of Helsinki and was approved by the Research Ethics Committee of University of Fukui (20200086). All participants signed a written informed consent form. From September 2020 to August 2023, peripheral venous blood samples were obtained from healthy controls and HCC patients at the University of Fukui Hospital. HCC was diagnosed radiologically in accordance with the American Association for the Study of Liver Diseases practice guidelines for the management of HCC, or histologically by needle biopsy of the liver tumor. ALBI (albumin-bilirubin) score was calculated using the following formula: (log10 bilirubin (μmol/L) × 0.66) + (albumin (g/L) × −0.085). ALBI grade was defined by the following scores: ≤–2.60 = Grade 1, >–2.60 to ≤ –1.39 = Grade 2, >–1.39 = Grade 3 [9]. Using reported cut-off value (−2.270), ALBI grade 2 was subdivided into 2 grades (2a and 2b) and named as modified ALBI grade [10].

### 2.2 Basal hepatic disease

The etiology was defined as HBV positive in patients with positive hepatitis B virus (HBV) surface antigen (HBsAg). Hepatitis C virus (HCV) positive was determined in patients with positive anti-HCV antibody (HCV Ab). Patients negative for both the HBsAg anti-HCV Ab and were defined as non-B-non-C (NBNC).

### 2.3 Treatment of HCC

Atezolizumab (1200 mg) and bevacizumab (15 mg/kg) were administered every 3 weeks according to the IMbrave 150 protocol until loss of clinical tumor progression or unacceptable and severe toxicity [3]. Atezolizumab and bevacizumab were discontinued or dose reduced to manage adverse events (AEs). Using gadolinium ethoxybenzyl magnetic resonance imaging (Gd-EOB-MRI) or dynamic computed tomography, radiological responses were evaluated with the Response Evaluation Criteria for Solid Tumors (RECIST) criteria. Conventional transcatheter arterial chemoembolization (cTACE) was performed by injecting an emulsion that contained 30–50 mg of miriplatin (MIRIPLA®; Dainippon Sumitomo Pharma, Osaka, Japan) and lipiodol (Lipiodol®; Guerbet Japan, Tokyo, Japan). Gelatin particles (Gelpart®; Nippon Kayaku Co., Ltd., Tokyo, Japan) were injected until adequate embolization was achieved. Drug-eluting beads TACE (DEB-TACE) was performed using drug-eluting beads (DC Beads®; Eisai Co., Ltd., Tokyo, Japan) with epirubicin, as previously described [11]. AEs were evaluated by the National Cancer Institute Common Terminology Criteria for Adverse Events version 5.0 (https://ctep.cancer.gov/protocoldevelopment/electronic_applications/ctc.htm).

### 2.4 Evaluation of the treatment response

Progression-free survival (PFS) was defined as the period from the start of treatment to disease progression or death, and overall survival (OS) was defined as the period from the start of treatment administration to death.

### 2.5 Enrichment of CTCs

Peripheral venous blood samples (10 mL each) were collected into ethylenediaminetetraacetic acid (EDTA)-2-Na anticoagulant tube. To avoid contamination by epithelial cells, the first 5 mL of blood was not used for CTC collection. CTCs were enriched using the RosetteSep Human CD45 Depletion Cocktail (StemCell Technologies, Vancouver, Canada). Blood samples were processed within 4 h after collection. After incubation with 50 μL Depletion Cocktail per 1 mL of blood for 20 min, the blood was diluted with an equal volume of Dulbecco's phosphate buffered saline (PBS; Gibco™, Waltham, MA, USA) and 2 % fetal bovine serum (FBS; Gibco™). The samples were then centrifuged at 1200×*g* for 15 min at RT using SepMate TM-50 mL tubes (StemCell Technologies) containing Lymphoprep™ (StemCell Technologies). The supernatant was transferred to a new standard tube and filled with buffer (PBS/2 % FBS). After centrifugation at 300×*g* for 10 min, cell pellets were suspended in PBS and collected.

### 2.6 Immunofluorescence staining

Enriched cells were incubated with APC/Cyanine7 mouse anti-human CD45 (BioLegend), PE mouse anti-human pan-Cytokeratin (Cayman Chemical), PE-Cy7 mouse anti-human CD90 (BD Biosciences), BV510 mouse anti-human CD133 (BD Biosciences), APC mouse anti-human EpCAM (BD Biosciences), Alexa Fluor 488 mouse anti-human Vimentin (BD Biosciences), or isotype control mouse IgG (BD Biosciences). The antibodies used are listed in Table 1. Cells were fixed with 4 % paraformaldehyde and the nuclei were stained with 4′-6-diamidino-2-phenylindole (DAPI) (PerkinElmer/Akoya Biosciences). Fluorescence microscope, BZ-X800 (Keyence, Japan), was used to detection the immunofluorescence. Combinations of fluorescent colors and filters (excitation and emission parameters) were described in Supplemental Tables 1 and 2

**Table 1:** Antiobodies used in this study.

Target	Application	Target species	Host species	Clone	Company	Catalogue No.
CD45	FCM; IF	Human	Mouse	2D1	BioLegend	368516
pan-Cytokeratin	FCM; IF	Human	Mouse	C-11	Cayman Chemical	10478
CD90	FCM; IF	Human	Mouse	5E10	BD Biosciences	561558
CD133	FCM; IF	Human	Mouse	W6B3C1	BD Biosciences	747644
EpCAM	FCM; IF	Human	Mouse	EBA-1	BD Biosciences	347200
Vimentin	FCM; IF	Human	Mouse	RV202	BD Biosciences	562338

Abbreviations: FCM, Flow cytometry; IF, Immunofluorescence.

### 2.7 Flow-cytometric analysis

Cells enriched with RosetteSep were labelled with APC/Cyanine7 mouse anti-human CD45 (BioLegend), PE mouse anti-human pan-cytokeratin (Cayman chemical), PE-Cy7 mouse anti-human CD90 (BD Biosciences), BV510 mouse anti-human CD133 (BD Biosciences), APC mouse anti-human EpCAM (BD Biosciences) and Alexa Fluor 488 mouse anti-human Vimentin (BD Biosciences). To detect dead cells, 7-aminoactinomycin D (7-AAD; BD Biosciences) was added to the buffer immediately prior to flow cytometry. In this study, we considered 7-AAD(−)CD45(−)PanCK(+) populations detected by flow cytometry to be CTCs. Flow cytometry was carried out on a BD FACS Area (BD Biosciences), and analyzed with FlowJo v10.10 (BD Biosciences). For compensation, isotype control IgGs(BD Biosciences) and CompBeads (BD Biosciences) were used. The used antibodies are listed in Table 1.

### 2.8 RNA extraction and nested-PCR

Total RNA was extracted from cells enriched with RosetteSep using an RNeasy Mini Kit (Qiagen, Hilden, Germany). cDNA was synthesized from RNA using a High-Capacity cDNA Reverse Transcription Kit (Applied Biosystems, Foster City, CA, USA) following the manufacturer's instructions. To increase the sensitivity of subsequent real-time PCR analysis, cDNA was pre-amplified in 14 PCR cycles with primers and the TaqMan Preamp Master Mix Kit (Applied Biosystems). To analyze mRNA expression, pre-amplified cDNA was evaluated using real-time PCR (StepOne Plus, Applied Biosystems). The primers and probes were obtained from Applied Biosystems (Table 2). The expression of target genes were analyzed using the ΔΔCt comparative threshold method. GAPDH was used as an internal control.

**Table 2:** Primers used in this study.

Gene	Species	Dye	Company	Catalogue No.
NANOG	Human	FAM	Applied Biosystems	Hs02387400_g1
POU5F1 (OCT3/4)	Human	FAM	Applied Biosystems	Hs04260367_gH
SOX2	Human	FAM	Applied Biosystems	Hs01053049_s1
CDH2 (N-cadherin)	Human	FAM	Applied Biosystems	Hs00983056_m1
GAPDH	Human	FAM	Applied Biosystems	Hs02786624_g1

### 2.9 Statistical analyses

The Wilcoxon matched pair rank test, Mann–Whitney *U* test, or Tukey–Kramer post hoc test were conducted to analyze the statistical significance. Cumulative survival was operated with the Kaplan–Meier method, and analyzed by the log-rank test. Statistical analyses were conducted by GraphPad Prism software v10 (GraphPad Software Inc., San Diego, CA, USA). The results were considered statistically significant at p < 0.05.

## 3 Results

### 3.1 Assay system for molecular biological characterization of circulating tumor cells

**Fig. 1:**
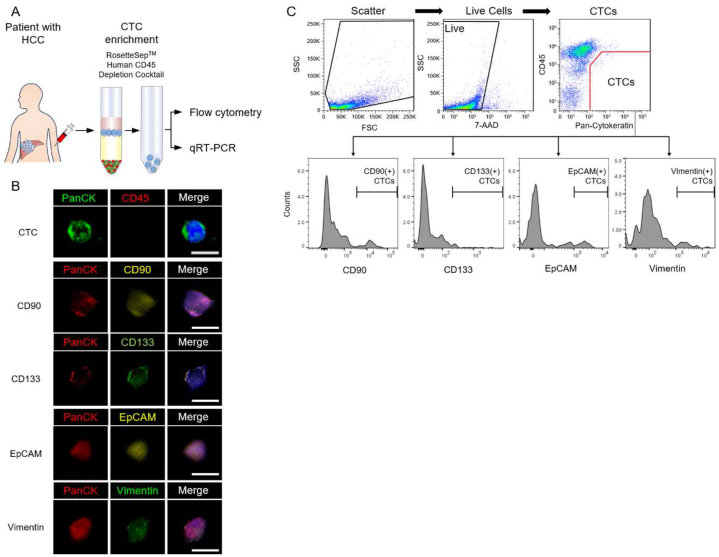
Assay system for molecular biological characterization of circulating tumor cells (CTCs) using multiparametric flow cytometry. (A) Peripheral blood of patients was collected and CTCs were enriched by Rosettesep. The expression of surface protein was then analyzed by flow cytometry. The gene expression was analyzed by qRT-PCR. (B) Immunofluorescence staining was performed in CTC. PanCK, CD45, CD90, CD133, EpCAM, vimentin and DAPI were stained. Representative images are shown. Bar, 10 μm. (C) Flowchart of CTC isolation and molecular analysis using multiparametric flow cytometry. (i) removing dead cells by Fixable Viability Stain, (ii) isolation of CD45(−) PanCK(+) cells as CTCs, and (iii) CD90, CD133, EpCAM, or vimentin positive cells.

To investigate the molecular characteristics of CTCs in patients with HCC, we constructed an assay system using multiparametric flow cytometry after CTC enrichment. Peripheral venous blood samples were collected from 10 healthy controls and 40 patients with HCC. The clinical characteristics are summarized in Table 3. To capture and enrich CTCs from peripheral blood, we used the RosetteSep™ Human CD45 Depletion Cocktail, which depletes white blood cells and red blood cells by density centrifugation with tetrameric antibody complexes recognizing CD45, CD66b, and glycophorin A (Fig. 1A). After CTC enrichment, PanCK(+) and CD45(−) tumor cells were observed by immunofluorescent staining (Fig. 1B). In addition, PanCK(+) CTCs showing markers CD90, CD133, EpCAM, or vimentin were observed. Enriched CTCs were then analyzed in three steps by flow cytometry: (i) removing dead cells, (ii) isolation of CD45(−) PanCK(+) cells as CTCs, and (iii) analysis of CTCs producing CD90, CD133, EpCAM, or vimentin, which are proteins associated with cancer progression (Fig. 1C). This CTC assay enables precise discrimination of CTCs in peripheral blood and the evaluation of their molecular biological characteristics in patients with HCC.

**Table 3:** Characteristics of patients with hepatocellular carcinoma and healthy controls.

Characteristics	HCC (n = 40)	Healthy controls (n = 10)
Age, median (IQR), years	73 (66–79)	70 (63–72)
Gender, male/female, n	28/12	7/3
Etiology, HBV/HCV/NBNC, n	4/15/21	–
PLT, × 10^9^/L, median (IQR)	144 (111–183)	257 (178–271)
PT, INR, median (IQR)	1.06 (0.98–1.15)	0.93 (0.89–0.97)
ALB, g/dL, median (IQR)	3.5 (3.1–3.8)	4.1 (3.8–4.2)
T-bil, g/dL, median (IQR)	0.9 (0.5–1.2)	0.8 (0.6–0.9)
ALT, IU/L, median (IQR)	27 (19–34)	18 (16–22)
AFP, ng/mL, median (IQR)	13.5 (4.5–241.2)	–
DCP, mAU/mL, median (IQR)	173 (27–1510)	–
Maximam tumor size, cm, median (IQR)	3.2 (2.1–6.9)	–
Number of tumor, 1/2/3+, n	9/6/25	–
Vascular invasion, absent/present, n	29/11	–
Extrahepatic metastasis, n		
None	33	–
Lymph node	3	–
Bone	1	–
Lung	1	–
Lung, Bone	1	–
Lymph node, Bone, Adrenal gland	1	–
BCLC stage, A/B/C, n	10/12/18	–
Treatments		–
Atezolizumab and Bevacizumab	21	–
Lenvatinib	3	–
Ramucirumab	1	–
TACE	15	–

Abbreviations: AFP, a-fetoprotein; BCLC stage, Barcelona Clinic Liver Cancer stage; DCP, des-gamma-carboxy prothrombin; IQR, interquartile range; NBNC, nonB-nonC; TACE, transarterial chemoembolization.

### 3.2 Number of CTCs expressing CD90, CD133, vimentin, and EpCAM molecules correlated with the BCLC stage

**Fig. 2:**
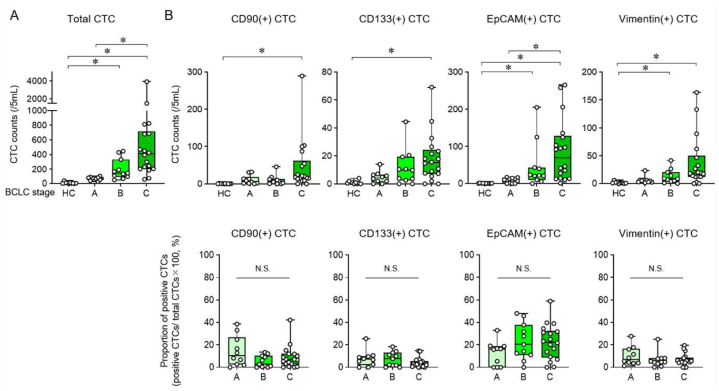
Analysis of CTC subtypes according to BCLC stage in HCC patients. (A) Total CTC counts in healthy controls (HCs) (n = 10) and HCC patients by BCLC stage (BCLC stage A/B/C; n = 10/12/18). (B) CD90, CD133, EpCAM, and vimentin-positive CTC counts in HCs and HCC patients by BCLC stage (upper panel) and percentage of each marker-positive CTCs to total CTCs in patients with HCC (lower panel). Centre lines of the box and whisker plot represent median values, whereas the box edges indicate the 25th and 75th percentiles, and the whiskers indicate the minimum and maximum values. Tukey–Kramer post hoc test. *p < 0.05; N.S., not significant.

The number and characteristics of CTCs of patients with HCC in each BCLC stage were evaluated. The total number of CTCs in healthy controls (HCs) was significantly lower than the number of CTCs in HCC patients with BCLC stages B and C (Fig. 2A). In HCC patients, the number of CTCs was consistent with BCLC stage progression, with a significant increase in stage C compared to patients in stage A. CD90, CD133, and vimentin(+) CTC counts were significantly increased in patients with stage C compared to HCs (Fig. 2B). The number of EpCAM(+) CTCs increased with the progression of BCLC stage. These results indicate that the total number of CTC and the number of CTCs expressing CD90, CD133, vimentin and EpCAM correlated with the BCLC stage of HCC patients.

### 3.3 Clinical course of CTC in representative patients with HCC treated with atezolizumab and bevacizumab therapy

**Fig. 3:**
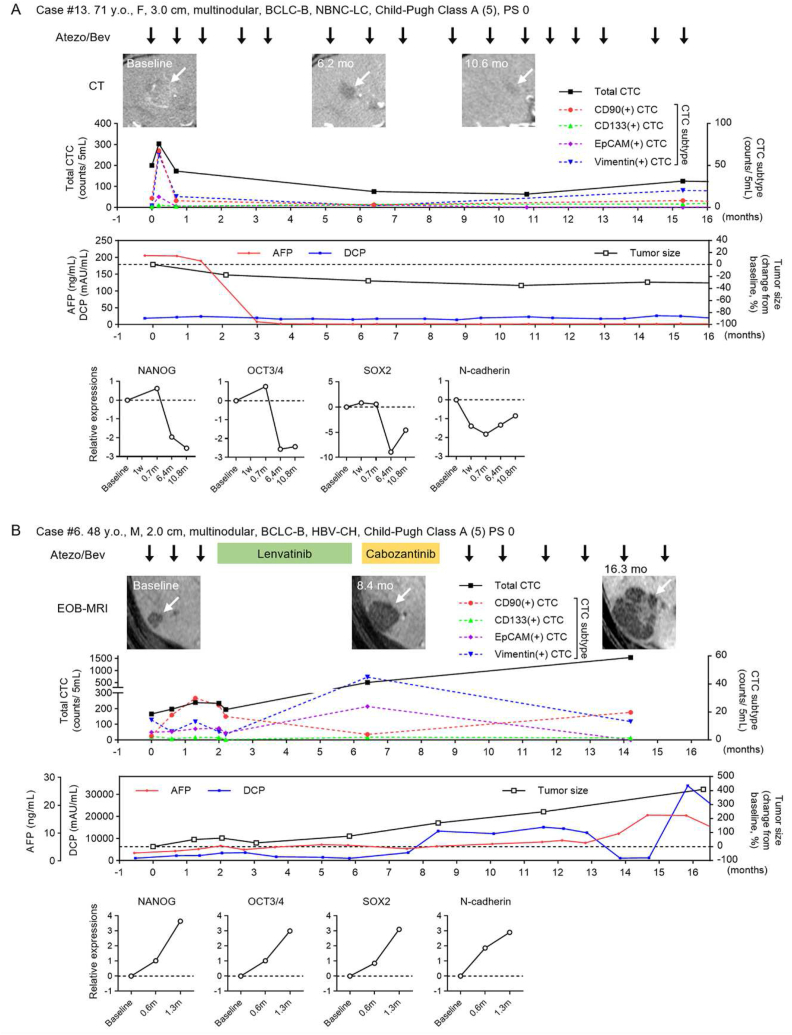
Clinical course of CTCs in representative HCC patients treated with atezolizumab and bevacizumab therapy. (A, B) Two representative clinical courses of patients treated with atezolizumab and bevacizumab (Atezo-Bev) for HCC. Case #13 achieved a partial response (PR) (A) and case #6 showed progressive disease (PD) (B) at the first treatment response assessment. The course of imaging studies [arterial phase of dynamic computed tomography (A), hepatobiliary phase of Gd-EOB-MRI (B)], total number of CTCs and CTC subtypes (CD90^−^, CD133-, EpCAM-, vimentin(+) CTCs), tumor marker (AFP, DCP) values, and RNA gene expression in the CTCs (NANOG, OCT 3/4, SOX2, N-cadherin).

The clinical course of CTC and the therapeutic response are shown in representative cases of HCC treated with atezolizumab plus bevacizumab (Atezo-Bev) (Fig. 3). The first patient (case #13, 71 years old, female) was complicated by multinodular HCC (Fig. 3A). The number of total CTC, CD90(+) and EpCAM(+) CTC decreased 0.7 months after starting Atezo-Bev therapy and decreased in subsequent courses. AFP levels decreased and the tumor size was reduced by 17.3 % at 2.1 months. Subsequently, the tumor exhibited a sustained partial response (PR). The expression of Nanog homeobox (NANOG), OCT3/4, SRY-box transcription factor 2 (SOX2), stem cell-associated genes, N-cadherin, and epithelial-mesenchymal transition (EMT)-associated genes expressed by CTCs was reduced by Atezo-Bev therapy.

The second case (case #6, 48-year-old, male) was complicated by multinodular HCC (Fig. 3B). The total CTCs and CD90(+) and EpCAM(+) CTCs were increased after 0.6 months of Atezo-Bev therapy. Tumor size increased by 61 % after 2.0 months. The number of total and subtypes of CTC increased after treatment with the multikinase inhibitors lenvatinib and cabozantinib and tumor size increased. The expression of NANOG, OCT3/4, SOX2, and N-cadherin was up-regulated in CTCs. These results suggest that the total number of CTCs and CD90(+) and EpCAM(+) CTCs may be accurate biomarkers of clinical response during the course of treatment of patients with HCC receiving Atezo-Bev therapy.

### 3.4 Total number of CTCs reflects the early treatment effect of Atezo-Bev therapy

**Fig. 4:**
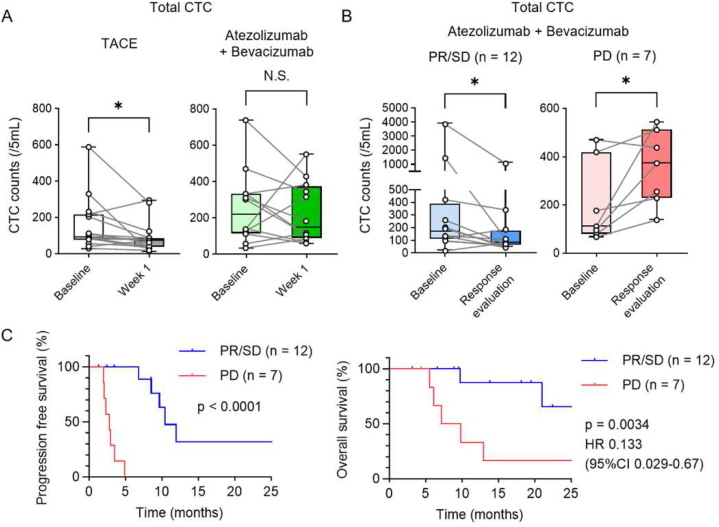
The change in the total number of CTCs at the first response assessment to Atezo-Bev therapy in patients with HCC. (A) The change of total number of CTCs at baseline and 1 week after treatment in HCC patients treated with TACE (n = 15) and Atezo-Bev therapy(n = 12). (B, C) The change of total CTC counts at baseline and at the time of first response evaluation in HCC patients treated with Atezo-Bev therapy who were PR/SD (n = 12) and PD (n = 7) (B). Kaplan–Meier curves of progression free survival and survival rate of these patients (c). (A, B) Centre lines of the box and whisker plot represent median values, whereas the box edges indicate the 25th and 75th percentiles, and the whiskers indicate the minimum and maximum values. Wilcoxon matched-pairs rank test. (C) Log-rank test. *p < 0.05, CI, confidence interval; HR, hazard ratio; N.S., not significant.

Changes in total CTC counts were investigated in patients with HCC treated with TACE or Atezo-Bev. The clinical characteristics are summarized in Table 4. After 1 week of treatment, the total CTC count was significantly reduced with TACE treatment but not with Atezo-Bev therapy (Fig. 4A). Among 40 patients with HCC, 21 patients were treated with Atezo-Bev. The change in the number of CTCs during Atezo-Bev therapy was evaluated in 19 patients, excluding 2 patients whose CTCs could not be collected at the time of the first treatment response evaluation. At the time of the first response evaluation to Atezo-Bev therapy according to RECIST 1.1, the change in total CTC counts was examined. In the PR/stable disease (SD) group, the total CTC counts decreased significantly (median CTC counts: baseline/response evaluation, 173.0/83.5 cells) (Fig. 4B). In patients with progressive disease (PD), the total number of CTC increased significantly (median CTC count: 112.0/374.0 cells). The PR/SD group in the first response evaluation had better PFS and OS than the PD group (Fig. 4C). These results indicate that the changes in total CTC counts could accurately reflect the initial response to Atezo-Bev therapy in patients with HCC.

**Table 4:** Characteristics of patients with hepatocellular carcinoma treated with TACE or atezolizumab + Bevacizumab.

Characteristics	TACE (n = 15)	Atezolizumab + Bevacizumab (n = 19)
Age, median (IQR), years	73 (71–76)	71 (64–79)
Gender, male/female, n	10/5	15/4
ECOG PS, 0/1/2/3/4, n	13/2/0/0/0	16/3/0/0/0
Etiology, HBV/HCV/NBNC, n	0/5/10	3/8/8
PLT, × 10^9^/L, median (IQR)	128 (101–162)	152 (116–176)
PT, INR, median (IQR)	1.08 (1.04–1.18)	1.06 (0.99–1.14)
ALB, g/dL, median (IQR)	3.4 (3.0–3.8)	3.5 (3.1–3.8)
T-bil, g/dL, median (IQR)	0.7 (0.5–1.1)	0.9 (0.6–1.3)
modified ALBI grade, 1/2a/2b/3, n	4/0/11/0	4/5/9/1
ALT, IU/L, median (IQR)	30 (24–33)	27 (21–46)
AFP, ng/mL, median (IQR)	5.3 (3.7–50.8)	15.1 (7.3–277.0)
DCP, mAU/mL, median (IQR)	85 (39–241)	640 (35–1511)
Maximam tumor size, cm, median (IQR)	2.3 (1.7–2.9)	3.5 (2.8–7.7)
Number of tumor, 1/2/3+, n	6/4/5	1/2/16
Vascular invasion, absent/present, n	13/2	14/5
Extrahepatic metastasis, n		
None	10	14
Lymph node	3	3
Bone	1	1
Lung	1	1
BCLC stage, A/B/C, n	11/1/3	0/9/10
Prior systemic therapy, n		
None	15	11
Sorafenib	0	1
Lenvatinib	0	4
HAIC	0	1
Lenvatinib, HAIC	0	2
Observation period, median, days	–	298

Abbreviations: AFP, a-fetoprotein; BCLC stage, Barcelona Clinic Liver Cancer stage; DCP, des-gamma-carboxy prothrombin; ECOG, Eastern Cooperative Oncology Group; HAIC, hepatic arterial infusion chemotherapy; IQR, interquartile range; NBNC, nonB-nonC; PS, performance status; TACE, transarterial chemoembolization.

### 3.5 CTCs that express the CD90 and EpCAM molecules reflect the early treatment effects and OS of Atezo-Bev therapy

**Fig. 5:**
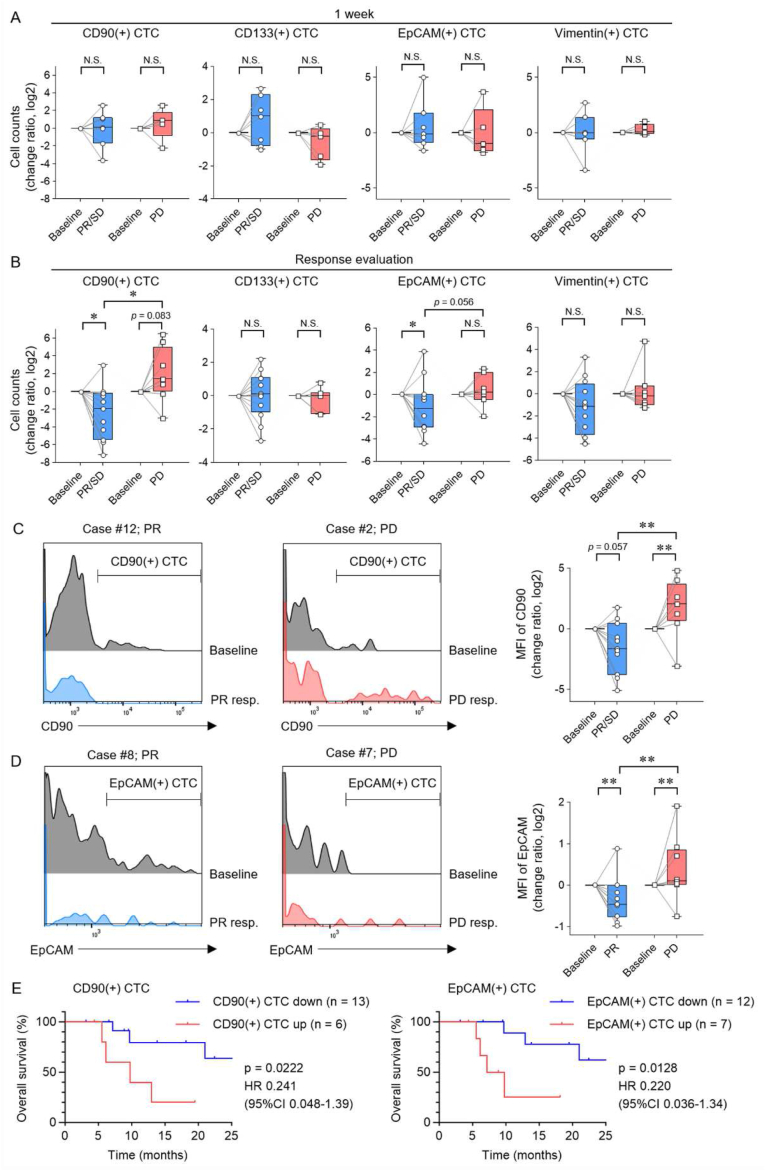
Analysis of association between molecular expression of CTCs, treatment response, and long-term prognosis in Atezo-Bev therapy in patients with HCC. (A-D) The change in CD90^−^, CD133-, EpCAM-, and vimentin (+) CTC counts at baseline and at 1 week after treatment initiation [PR/SD (n = 7) and PD (n = 5)] (A) and at the time of first response evaluation (B-D) in HCC patients treated by Atezo-Bev therapy who achieved PR/SD (n = 12) and PD (n = 7). (C, D) Fluorescence intensity (FI) of CD90 (C) and EpCAM (D) in CTC. The fluorescent intensity at baseline and first response evaluation in representative PR (left) and PD (middle) cases. The change in mean fluorescent intensity (MFI) at baseline and at the time of first response evaluation (right). (E) Kaplan-Meier curves of survival rate in patients with decreased or increased CD90(+) (left) or EpCAM(+) (right) CTC counts at initial response to treatment. (A-D) Centre lines of the box and whisker plot represent median values, whereas the box edges indicate the 25th and 75th percentiles, and the whiskers indicate the minimum and maximum values. (A-D) Mann-Whitney test. (E) Log-rank test. *p < 0.05, **p < 0.01, CI, confidence interval; HR, hazard ratio; N.S., not significant.

The association between changes in the number and characteristics of CTCs that express CD90, CD133, EpCAM, and vimentin during Atezo-Bev therapy and therapeutic response was analyzed in patients with HCC. In patients with PR/SD and PD as the initial response evaluation to Atezo-Bev, there was no change in the number of CTCs expressing CD90, CD133, EpCAM, and vimentin after 1 week (Fig. 5A). The numbers of CD90(+) and EpCAM(+) CTCs decreased in the PR/SD group and tended to increase in the PD group (Fig. 5B). The number of CD133(+) and vimentin (+) CTCs was not associated with the therapeutic response. Changes in the mean fluorescence intensity (MFI) of CD90(+) and EpCAM(+) CTCs were analyzed. The MFI of CD90(+) and EpCAM(+) CTCs were reduced in the PR/SD group and increased in the PD group (Fig. 5C and D). Patients with increased CD90(+) and EpCAM(+) CTC during Atezo-Bev therapy had significantly poorer OS than those with decreased CD90(+) and EpCAM(+) CTC [CD90(+)CTC: HR 0.241; 95%CI 0.048–1.39; p = 0.02, EpCAM(+)CTC: HR 0.220; 95%CI 0.036–1.34; p = 0.01] (Fig. 5E). These results indicate that the changes in the number and MFI of CTCs expressing CD90 and EpCAM reflect the initial therapeutic response and OS in patients with HCC treated with Atezo-Bev.

## 4 Discussion

In this study, we isolated CTC from the peripheral blood of patients with HCC and monitored their molecular and cellular characteristics during ICI therapy. The total number of CTCs and the number of CTCs expressing CD90, CD133, vimentin, and EpCAM were correlated with the BCLC stage of HCC patients. Changes in total CTC counts accurately reflected the initial response of the treatment to Atezo-Bev. Furthermore, the number and MFI of the CTC subsets expressing CD90 and EpCAM decreased in patients with PR/SD and increased in patients with PD, and were markedly correlated with OS of the patients after treatment. Collectively, CD90(+) and EpCAM(+) CTCs may be candidate biomarkers for the early prediction of treatment response and OS in patients with HCC treated with Atezo-Bev.

Unlike conventional cytotoxic chemotherapy, ICIs may require a longer period to elicit a response, and some patients experience atypical radiological features, including delayed and dissociated responses [12]. The RECIST criteria of responses to treatments in solid tumor patients relies primarily on unidimensional measurements of tumor size [13] and typically occurs at predefined time points, which may not capture the delayed responses seen with immunotherapy [14]. This can lead to an underestimation of the efficacy of treatment with ICIs, due to the premature timing of therapeutic evaluation.

Liquid biopsy has emerged as a noninvasive diagnostic method for detecting and monitoring cancers, including HCC [15]. It involves the analysis of circulating tumor-derived materials, such as extracellular vesicles (EVs), circulating tumor DNA (ctDNA), and CTCs in blood samples [16]. Recent studies have identified specific genetic alterations in ctDNA samples from patients with HCC, including mutations of the TP53 and CTNNB1 genes and TERT promoter mutations [17,18]. EV-based liquid biopsy holds promise for the diagnosis of HCC, as it allows the detection of tumor-specific molecules, such as proteins, RNAs and DNA [19]. CTCs originating from solid tumors are related to hematogenous metastatic spread to distant sites, and patients with CTC-positive HCC have been reported to have a higher risk of recurrence and shorter recurrence-free survival [20]. In addition, tumor cell dissemination may be an early event in the pathogenesis of HCC, as CTCs have been detected in 90 % of patients with HCC and more than half of patients with early stage HCC [21]. Consistently, CTCs were detected in a high percentage of patients with HCC in this study.

Molecular expression analysis of CTCs using flow cytometry is clinically relevant to the prognosis and therapeutic efficacy of colorectal cancer [22,23], prostate cancer [24], and ovarian cancer [25,26]. In this study, cell surface molecules of CTCs were stained with CD45 and CK antibodies as reported previously [[27], [28], [29]], and 7AAD-/CD45-/Pan CK + cells were determined as CTCs. However, flow cytometry must take into account technical difficulties in distinguishing between genuine CTCs and false positive events [25]. The number of CTCs counted in this study was higher compared to the CTC studies of HCC counted by fluorescence microscopy [8,21]. Since the number of CTCs in the HC was low in this study, it is possible that non-specific false bindings were counted as false positives in flow cytometry. Considering these possibilities, the protein expression in CTCs in patients with HCC was observed using fluorescence microscopy.

The clinical significance of molecular expression associated with EMT and Stem cell markers in CTCs has been reported [30,31]. Analysis of CTCs expressing vimentin [21] as a mesenchymal marker and CD133 [21], CD90 [21,[32], [33], [34]], and EpCAM [21,33,35] as stem cell markers in HCC has been reported to be useful for early diagnosis, prognosis prediction, and determination of therapeutic efficacy. Vimentin migrates from intracellular regions to the cell surface during EMT to produce cell surface vimentin [36,37]. Cell surface vimentin has been used as a target to collect mesenchymal CTCs and has been reported to be an effective diagnostic and prognostic biomarker for gastric [38], colorectal [36], pancreatic [39], lung [40], and breast cancers [41]. In this study, the clinical significance of CTCs expressing CD90, CD133, EpCAM, and vimentin in the course of Atezo-Bev was evaluated by serial collection of CTCs. Therapeutic effects were correlated with changes in CD90 and EpCAM expression observed in CTC at early time points of treatment in patients with HCC. CD90 is a marker of cancer stem cell (CSCs) that exhibits a comparable tendency in the association between circulating CSCs and clinical outcomes [42]. CD90(+) HCC cells have been shown to have high lung metastatic potential [43]. Gene expression profiling of CD90(+) HCC cells showed increased gene expression associated with inflammation, drug resistance, and cell proliferation [44]. These support the increased number of CD90(+) CTCs in patients who progressed during Atezo-Bev therapy in this study. Consistent with current results, EpCAM(+) CTCs reportedly correlate with a poor outcome in patients with metastatic non-small cell lung cancer, prostate cancer, and HCC [45,46]. A key event in the intravasation of HCC cells into the bloodstream is the EMT. EMT is a process in which epithelial tumor cells lose their adhesion capacity due to downregulation of EpCAM expression [47,48]. To extravasate circulating EpCAM downregulated CTCs from the bloodstream and form metastases, they must become EpCAM positive again through the mesenchymal epithelial transition, the reversal of EMT [49,50]. EpCAM(+) HCC cells are induced by activation of Wnt/β-catenin signaling [51]. Activation of Wnt/β-catenin signaling in HCC has been reported to be associated with resistance to ICIs [52]. These support the number and MFI of EpCAM(+) CTCs increased in patients with PD treated with Atezo-Bev.

There are several limitations to this study. First, although we performed the assay system using multiparametric flow cytometry after CTC enrichment in patients with HCC, only 19 patients received atezolizumab and bevacizumab treatment, which is a small fraction to draw definitive conclusions about the usefulness of CD90(+) and EpCAM(+) CTCs as predictive biomarkers of response to therapy. However, in these cases, CTCs were collected and evaluated at many points during the long-term course of Atezo-Bev therapy and correlated well with the image response determination of HCC. Second, this study was performed at a single institution. In the future, it may be important to increase the number of samples and analyze the molecular biological significance of CD90(+) CTCs and EpCAM(+) CTCs in Atezo-Bev therapy of HCC.

In conclusion, changes in the number and expression levels of CD90 and EpCAM molecules in CTCs at the early time point of Atezo-Bev therapy correlated with treatment effects and reflected in the long-term prognosis of HCC patients. Non-invasive monitoring of molecular alterations in CTC can contribute to the early detection of anticipated therapeutic responses in HCC patients treated with the immune checkpoint inhibitor combination therapy.

## 5 Ethics statement

This retrospective study was performed in accordance with the Declaration of Helsinki and was approved by the Research Ethics Committee of University of Fukui (20200086). Written informed consent was obtained from all participants. All participants/patients provided informed consent for the publication of their anonymised case details and images.

## Funding sources

This research was partially supported by 10.13039/100009619Japan Agency for Medical Research and Development (10.13039/100009619AMED) under Grant Numbers JP23fk0210113 and JP23fk0210104 and 10.13039/501100001691Japan Society for the Promotion of Science (10.13039/501100001691JSPS) 10.13039/501100001691KAKENHI Grant-in-Aid for Scientific Research Number 22K15992.

## Data availability statement

The data associated with this study have not been deposited in a publicly available repository. Data will be made available on request.

## CRediT authorship contribution statement

**Takuto Nosaka:** Writing – original draft, Visualization, Validation, Resources, Project administration, Methodology, Investigation, Funding acquisition, Formal analysis, Data curation. **Yosuke Murata:** Resources, Investigation. **Yu Akazawa:** Resources, Investigation. **Kazuto Takahashi:** Resources, Investigation. **Tatsushi Naito:** Resources, Investigation. **Hidetaka Matsuda:** Resources, Investigation. **Masahiro Ohtani:** Resources, Investigation. **Yasunari Nakamoto:** Writing – review & editing, Writing – original draft, Visualization, Validation, Supervision, Project administration, Methodology, Investigation, Funding acquisition, Data curation, Conceptualization.

## Declaration of competing interest

The authors declare that they have no known competing financial interests or personal relationships that could have appeared to influence the work reported in this paper.
