# Supplementary material for: Changes of circulating tumor cells expressing CD90 and EpCAM in early-phase of atezolizumab and bevacizumab for hepatocellular carcinoma
Source: Heliyon. 2024 Jul 10;10(14):e34441. doi: 10.1016/j.heliyon.2024.e34441 (PMC11301359; doi:10.1016/j.heliyon.2024.e34441)
Supplement: Multimedia component 1 [file mmc1.docx]

**Supplemental Table 1.** Filters used for fluorescence microscopy

| Filter | Excitation (nm) | Emission (nm) |
| --- | --- | --- |
| DAPI | 360 | 460 |
| GFP | 470 | 525 |
| Cy3 | 545 | 605 |
| Cy5 | 620 | 700 |

**Supplemental Table 2.** Combination of fluorescent antibodies and filters

| Target antibodies (fluorophore)  / Filter | DAPI | GFP | Cy3 | Cy5 |
| --- | --- | --- | --- | --- |
| DAPI, CD45, and PanCK | DAPI |  | PanCK  (PE) | CD45  (APC/Cyanine7) |
| DAPI, CD90, and PanCK | DAPI |  | PanCK  (PE) | CD90  (PE-Cy7) |
| DAPI, CD133, and PanCK | DAPI | CD133  (BV510) | PanCK  (PE) |  |
| DAPI, EpCAM, and PanCK | DAPI |  | PanCK  (PE) | EpCAM  (APC) |
| DAPI, Vimentin, and PanCK | DAPI | Vimentin  (Alexa Fluor 488) | PanCK  (PE) |  |
